# Role of Radiotherapy in Modern Treatment of Hodgkin's Lymphoma

**DOI:** 10.1155/2011/258797

**Published:** 2010-10-24

**Authors:** Kheng-Wei Yeoh, N. George Mikhaeel

**Affiliations:** Department of Clinical Oncology, Guy's and St. Thomas' Hospital, London, UK

## Abstract

Hodgkin's Lymphoma was incurable until the advent of effective therapeutic radiation around the first half of the 20th century. As survival rates improved, the long-term toxicities from radiotherapy began to emerge. This together with the availability of effective chemotherapy has encouraged a combined modality approach for early-staged disease and the omission of radiotherapy in advanced-staged disease. The differing toxicities of radiotherapy and chemotherapy has promoted ongoing research to identify the utility of each of these modalities in the modern management of Hodgkin's Lymphoma. This article will provide a critical review of the developments and indications for modern radiotherapy, in context with advances in chemotherapy, for the treatment of Hodgkin's Lymphoma.

## 1. Introduction

During the first half of the 20th century, Hodgkin's Lymphoma (HL) was incurable until the development of effective therapeutic radiation [1]. It involved irradiating beyond the site of disease to include the adjacent nodal groups, the so-called extended field radiotherapy (EFRT) with total or subtotal nodal irradiation [2]. With Radiotherapy (RT) alone, 10-year relapse-free survival of around 70% had been achieved in early-staged HL [3, 4]. 

However, advanced stage HL remained less curable with RT even with subtotal or total nodal RT until the introduction of combination chemotherapy. With the advent of effective chemotherapy regimes in the 1980s with MOPP [5] and ABVD [6], better cure rates were achieved in advanced disease establishing chemotherapy as the main line of treatment in advanced disease. Gradually, chemotherapy use moved to earlier-stage disease, initially as a pre-RT treatment in bulky mediastinal cases to reduce the extent of mediastinal RT and later to improve the outcome of RT alone. With emerging reports of long-term toxicities from EFRT, namely with pulmonary, cardiac toxicity, and secondary malignancies [7–13] together with studies showing that limited RT was sufficient after prior chemotherapy, combined modality treatment has been established in early disease. More recently, there has been several opinions calling on treating early stage HL with chemotherapy alone without RT, and this remains an interesting research question. 

The paper below will aim to highlight the developments of RT in HL treatment, with specific focus on its role in early, advanced, and relapsed disease.

## 2. Developments in RT

### 2.1. Background

The concept of RT to cure HL by Gilbert [14] was implemented with kilovoltage [15] and later with megavoltage machines [1]. Doses of up to 4500 rads (1000 rads/wk) were commonly used to treat extended fields in order to achieve total lymphoid irradiation (TLI). This included mantle field, inverted-Y techniques, and modifications to include splenic irradiation, delivered with a parallel opposed technique. It was also not uncommon to irradiate liver and lungs involved with disease; all this was done when staging laparotomy and splenectomy were standard practice. 

In the light of RT toxicities, suboptimal relapse and survival rates together with the introduction of effective chemotherapy, the impetus to rationalise RT delivery became a priority. This was achieved through prognostic risk stratification, combined modality approach with chemotherapy in concert with advances in RT. These include improvements with RT techniques and rationalisation in both the irradiated volume as well as the RT doses used. 

Risk stratification was introduced in the 1980s originally by the BNLI group [16], and also the EORTC and GHSG groups to rationalise treatment for good prognostic-HL patients in order to minimise toxicity. The EORTC/GELA and GHSG groups have identified several features indicative of unfavourable prognosis [17, 18] (Table 1) in early stage HL which has allowed studies to investigate risk stratified treatment. 

Advanced disease is classified as stage 3 and 4 disease and also includes stage 2B disease in presence of bulky mediastinal or extranodal disease in the GHSG classification.

### 2.2. RT Doses

From the traditional doses of over 40–45 Gy used, doses for treatment of HL have been rationally reduced in order to maintain optimal outcome with reduced toxicities. Doses of 30 Gy have been shown to yield similar results to higher doses used with no increased dose-response relationship seen for higher doses used [19–21], and as such 30 Gy in combined modality treatment (CMT) has become the standard in most countries. Lower doses have also been shown to be effective from 2 current studies, one by the EORTC/GELA group and the other the GHSG group. The ongoing EORTC/GELA H9F trial had 20 Gy Involved Field RT (IFRT) in one trial arm which showed similar results to higher doses of radiotherapy in combined modality treatment of early-staged HL [22]. Incidentally, the arm where RT was omitted has been stopped due to higher relapse rates found. Preliminary results from the ongoing GHSG HD10 trial has shown that dose reduction to 20 Gy IFRT as part of combined modality treatment showed equivalent results to higher doses [23].

### 2.3. RT Volume

As well as the ongoing reduction in RT doses being used, smaller RT fields have been used and several randomised trials have shown that IFRT, where only the nodal region is irradiated, is as effective as EFRT with equivalent overall survival rates [24–27]. Further progress has gone beyond the concept of IFRT to Involved Site RT (ISRT) and now Involved Node RT (INRT), where only the involved nodes are irradiated, have been proposed by the EORTC/GELA group [28]. Encouraging results have come from a retrospective series showing equivalent progression-free survival (PFS) and overall survival (OS) for INRT compared to IFRT [29].

### 2.4. RT Technology

In concert with the above developments, there has been significant improvement in RT techniques in order to achieve more conformal radiotherapy treatment from the traditional large parallel opposed fields used. This has allowed RT to progress to 2D conformal [30] and to now commonly used 3D conformal techniques [31], with Intensity-modulated RT (IMRT) being shown to be able to further improve target volume conformity with reductions of dose to critical structures [32]. These have therefore allowed the developments of reduction in irradiated volume as described above.

## 3. Role of RT in Early Disease

The improvement of clinical staging with the introduction of the Cotswolds modifications of the Ann Arbour staging in 1989 [33] together with adverse outcomes being reported with the routine use of staging laparotomy and splenectomy with no clinical benefit [16] led to recommendations to abandon these procedures. Clinical risk stratification identifying favourable and unfavourable groups within early-staged HL by groups such as the EORTC and GHSG has allowed investigation to rationalise treatment in early HL further. 

Results from several trials support CMT for the treatment of early-staged HL. 

Studies comparing CMT with RT alone have shown superiority of CMT. These have been shown by both the GHSG and EORTC groups. In the EORTC H8F trial, patients with early-staged favourable HL were randomised to either 3 cycles of MOPP/ABV chemotherapy followed by IFRT of 36 Gy or to RT alone with STNI. The results showed a better event-free survival (EFS) and OS in favour of the CMT arm (EFS 98% versus 74%, 10 yr OS 97% versus 92%) [26]. These results are supported by the GHSG HD7 trial favouring 2 cycles of ABVD chemotherapy followed by RT versus RT alone in early-staged favourable HL [34]. 

Studies comparing CMT with chemotherapy alone have shown that chemotherapy alone yields worse outcomes. The EORTC/GELA H9F trial compared 6 cycles of chemotherapy with or without IFRT [22]. The arm without RT was closed early due to higher-than-expected relapse rates. Similar findings were reported in the CCG 5942 trial comparing 4–6 cycles of chemotherapy with or without RT. The arm without RT was closed early due to a significantly higher number of relapses seen on the no-radiotherapy arm [35]. Two North American studies comparing chemotherapy alone versus CMT showed an inferior PFS and freedom from progression (FFP) in the chemotherapy alone arms with the NCIC/ECOG HD-6 Trial showing a 5-Year PFS of 87% versus 93%, in favour of CMT [36] and the MSKCC trial showing a FFP of 86% versus 81% [37] in favour of CMT. Advantages in overall survival for CMT were shown in the Tata Memorial Trial which showed an 8 year EFS of 88% versus 76% and OS of 100% versus 89% in favour of CMT versus 6 cycles of ABVD alone [38]. Although all stages of HL were included in this trial, 55% of enrolled patients had early HL.

Based on these results and data supporting ABVD over other combination chemotherapy regimes [39–42], the recommendations for the treatment of early HL are with the combined modality approach with 2 to 4 cycles of ABVD followed by IFRT of 30 Gy [43, 44] with the ESMO group making further recommendations that favourable early HL patients should receive 2 cycles of ABVD in combination with IFRT whilst 4 cycles of ABVD in combination with IFRT being reserved for the unfavourable group. Further dose de-escalation studies for favourable early HL are underway such as the NCRN RAPID trial in the UK using information from interval PET imaging. The prognostic information from interval PET scanning in HL [45] has also formed the basis for the GHSG HD16 trial and EORTC 10F trial. The use of PET imaging is also now becoming standard for routine staging of HL [46] as well as gaining support for use in RT planning with INRT [47].

Some controversy has arisen over the proposition for the use of chemotherapy alone in the management of early HL [48–50] lending support for this approach to be within an option of the NCCN guidelines for the management of early HL [51]. The impetus for this approach was based on toxicity data during the era where EFRT was routine. There remains no evidence to support this approach with worse reported outcomes when RT is omitted, as highlighted from the various studies discussed earlier. A recent meta-analysis studying 5 randomised control trials [52] has confirmed the superiority of CMT over chemotherapy alone with a hazard ratio (HR) of 0.41 for tumour control and 0.40 for OS for patients receiving CMT compared to chemotherapy alone. As such the standard of care for early HL remains CMT.

A caveat for radical treatment with radiotherapy alone is with Nodular Lymphocyte Predominant Hodgkin's Lymphoma (NLPHL) where 30 Gy has been shown to yield similar results compared with combined-modality treatment for Stage 1A NLPHL with no risk factors whilst advanced NLPHL has been treated with classic HL protocols [53, 54].

## 4. Role of RT in Advanced Disease

In the prechemotherapy era, advanced HL was treated with Total Lymph Node Irradiation (TLI) where the majority of patients relapsed and died. With the advent of MOPP, long term remission of around 50% were achieved [55]. This was then replaced by ABVD after randomised trials showed superiority over other regimes including MOPP [56, 57]. Several other regimes have also been shown to be more toxic but no more effective than ABVD [39–42, 58] with 3-year OS survival rates of around 90% being achieved. Based on these findings, ABVD has now become the standard arm for comparison in ongoing trials. But with suboptimal long-term failure-free and survival rates, dose escalated regimes have been used to improve results [59], however not without increased toxicities. Direct comparison of escalated BEACOPP with the standard ABVD regime is ongoing with the EORTC 20012 trial. 

The Stanford V protocol offered an alternative regime with a short period of 12 weeks of chemotherapy followed by RT to bulky sites, classified as 5 cm or more [60]. However, comparison of the Stanford V regime with ABVD and MOPPEBVCAD showed worse PFS [61]. Results are awaited for the UK NCRI Stanford V versus ABVD trial for advanced HL.

Offering RT after effective chemotherapy is not standard practice and is still undergoing investigation. However, data so far can allow risk stratification into groups whom may benefit. Although a meta-analysis [62] and studies by the GELA [63] and EORTC groups [64] showed no benefit of consolidation RT after effective chemotherapy with suggestions of worsened outcome when RT was added, more recent data have emerged from 2 large randomised control trials (RCT) in support of consolidation RT. The UKLG LY09 trial showed 5 year PFS of 71% versus 86%, with OS HR of 0.47 in favour of consolidation RT [65]. This concurs with another large RCT from Tata Memorial Hospital showing EFS and OS advantage for consolidation RT after 6 cycles of ABVD chemotherapy in all stages of HL [38]. Furthermore, most of the trials of chemotherapy dose escalation include RT for most patients in their protocols. Utilising the extra data obtained from PET to identify metabolically active residual masses postchemotherapy, the GHSG HD15 trial incorporates PET to assess residual lesions >2.5 cm to receive consolidation RT, and preliminary results have been able to show favourable negative predictive value of PET of 94% [66] hence offering the potential towards a risk stratified approach.

## 5. Role of RT in Salvage Treatment

Although the standard treatment for patients with refractory and relapsed HL to chemotherapy and combined chemo-radiotherapy is high-dose therapy supported by autologous stem-cell transplant, RT has a role in the peritransplantation setting. Results by Poen et al. at Stanford [67] has shown that most relapses occurred in areas of known disease pretransplant and posttransplant irradiation resulted in no in-field relapses. This concept has been supported by a series at MSK showing favourable results [68, 69]. Results from clinical trials in this setting as well as studies looking at the utility of PET to identify peritransplant active disease should yield exciting recommendations.

## 6. Conclusion

From the above review, it can be seen that the role of RT in the management of HL has changed considerably over the last century with the advent of better staging, risk stratification strategies, effective chemotherapy and salvage regimens, and the progress in RT techniques and technology. RT doses and volume have been significantly rationalised to minimise toxicities with the establishment of highly conformal RT techniques whilst allowing concepts such as INRT to be introduced. 

It is important to highlight that studies which have shown toxicities with RT have been based on historical series where obsolete nonconformal RT with higher doses were used and that survival data reported in trials may be misrepresented with the prevalence and success rates of salvage chemotherapy regimes and transplantation in relapsed HL. 

In early-staged HL, CMT is thus the established standard of care with adjustments in the number of chemotherapy cycles justified according to the risk factors. In advanced-staged HL, a full course of chemotherapy is the standard with special circumstances advocating consolidation RT, namely initial bulk disease or residual PET avidity. Ongoing trials will no doubt clarify the role of RT in these situations as well as identifying the role of PET to dictate consolidation RT.

Ongoing work with the above strategies of achieving long-term cure whilst minimising toxicities, whether it be from radiotherapy or chemotherapy, should continue to yield promising results.

## Figures and Tables

**Table 1:** EORTC/GELA and GHSG risk factors for early-staged HL.

Early-staged (I-II) unfavourable features (EORTC/GELA)	Early-staged (I-II) unfavourable features (GHSG)
Four or more nodal areas involved	Three or more nodal areas involved
Bulky mediastinum*	Bulky mediastinum*
ESR >50 without B symptoms or >30 with B symptoms	ESR >50 without B symptoms or >30 with B symptoms
Aged 50 years old or more	Extranodal disease

*Bulky mediastinum defined as mediastinal/thoracic ratio >0.35.
